# Factors Related to Small- and Mid-Capitalization Pharmaceutical Company Success Using Stock Performance as a Surrogate

**DOI:** 10.7759/cureus.18210

**Published:** 2021-09-23

**Authors:** Matthew J Ferris, Kai Sun, Corey Savard, Tejas Suresh, Mark V Mishra

**Affiliations:** 1 Radiation Oncology, University of Maryland School of Medicine, Baltimore, USA; 2 Radiation Oncology/Statistician, University of Maryland School of Medicine, Baltimore, USA; 3 Internal Medicine, Lankenau Medical Center, Wynnewood, USA; 4 Medical Oncology, Virginia Cancer Specialists, Fairfax, USA

**Keywords:** drugs, therapeutics, drug development, stocks, biotech, pharmaceuticals, drug pricing

## Abstract

Background

Developing novel pharmaceuticals demands substantial investment despite high uncertainty of success and ultimate market value. While many established drug companies are highly profitable and have large portfolios of diversified assets, much of new drug innovation, a very high-risk, high-reward gambit, stems from smaller companies striving to bring their first products to market. While drug costs, and thus pharmaceutical company profits, can be controversial, it is unquestionable that the products from these companies provide great benefit to humanity. Hence, the ongoing success of the industry as a whole is quite relevant from a public health perspective.

Methodology

We sought to investigate factors influencing pharmaceutical company success using company stock performance on major US indices as a surrogate. As the profitability of large-capitalization (cap) pharmaceutical companies is well established, we focused on small- and mid-cap companies in this analysis. Small- and mid-cap pharmaceutical companies (both currently active and now defunct) and historical share prices were captured, including company details and the nature of drug pipelines. Funding by US academia was acquired via CMS.gov Open Payments and categorized into contributions < or ≥$100,000. Stock performance was considered good (+ ≥25%), mediocre (±25%), or poor (- ≥25%). Univariate and multivariate associations were assessed.

Results

Of the 420 companies included in the analysis, 101 (24%) had good, 76 (18%) mediocre, and 243 (58%) poor performance. The following were associated with performance in univariate analysis: initial public offering (IPO) price (*P* < 0.001), time from IPO (*P* < 0.001), number of drug programs (*P* = 0.019), and academic funding (*P* = 0.00013), with trend for diverse pipelines (both oncology and nononcology programs under development) (*P* = 0.069). On multivariate analysis, IPO price was inversely associated (*P* < 0.0001), while academic funding (*P* < 0.0001) and more drug programs (*P* = 0.0025) were positively associated with performance. Analysis of pharmaceutical IPOs since 2000 suggests a 20% rate of outright company failure.

Conclusions

The majority of included companies had lackluster stock performance, suggestive of low potential for drug development success and high probability of financial disaster. Robust drug pipelines and academic collaboration seem to be strongly related to company success.

## Introduction

Developing novel pharmaceuticals demands substantial investment [1-3]. Despite high upfront research and development costs, there remains great uncertainty in eventual drug approval and market value. Over 10 years and approximately a billion dollars are the commonly cited time and cost required to bring a drug to market. In this process, numerous preclinical candidates are first narrowed to the few most promising, followed by in vivo studies in animal models, which may progress to first-in-human studies, safety and efficacy studies, and, finally, to the randomized placebo-controlled trials that are often required to supply enough evidence for approval by the Food and Drug Administration (FDA) [2]. As companies have 20 years from drug patenting until generic competition may emerge, the duration for which novel drugs may provide optimal profits might be less than 10 years.

High drug costs have become subject to recent controversy and politics in the United States, where per-capita prescription drug spending exceeds all other countries [4-9]. A recent report described the higher relative profitability of large pharmaceutical companies compared to other large public companies; hence, clearly, successful drugs do yield a financial windfall [10]. While the profitability of large market capitalization (cap) pharmaceutical companies (e.g., worth over $10 billion) is now appreciated, much of the early innovation is not performed in-house at these companies. Instead, a multitude of small- and mid-cap (worth less than $10 billion) companies, which can have a tighter focus than a pharmaceutical giant, embark on the high cash-burn business of shepherding innovative drug assets through early development, with the ultimate goal of lucrative return on investment years later. Established large caps can utilize cash reserves for licensing and partnership with these smaller companies or perform outright mergers and acquisitions; often acquisition transpires once early-stage assets are de-risked and the likelihood of drug approval is high or has already transpired. This process works reasonably well for large caps as they skirt much of the early financial risk when uncertainty is the highest. For smaller companies, it tends to be feast or famine. While there is a high likelihood of failure, success often brings either millions of dollars in revenues from approved drugs or a premium on the initial development expenditures brought with an acquisition by a larger company.

Because the profitability of large pharmaceutical companies is well established, we sought to analyze factors influencing success for small- and mid-cap companies, which initiate the development of a critical portion of the novel drugs eventually available for public use. Stock performance over time on major US stock indices was used as a surrogate for company success. A subset analysis focused on companies that specialize in oncology, given the often-panned price points of new oncology drugs [1,11-17].

## Materials and methods

A stock screener (Yahoo! Finance, Verizon Media, Sunnyvale, CA, USA) and a comprehensive list of historical initial public offerings (IPOs) (IPOScoop, IPOScoop.com, Rahway, NJ, USA) were utilized to capture active publicly traded small- and mid-cap drug companies with a share price of ≥$1.00 on either the New York Stock Exchange (NYSE) or the Nasdaq Stock Market (Nasdaq). The $1.00 cutoff was used because stocks trading under $1.00/share are deemed to have slim prospects for future success, are at a risk for extreme volatility, and if under $1.00/share for 20 consecutive days are at risk of delisting from the NYSE. To allow for passable follow-up time, companies that underwent IPO in 2019 or later were excluded.

Details of the active companies and the nature of their drug pipelines were obtained from company websites, supplemented via a database of business information, Crunchbase (Crunchbase Inc., San Francisco, CA, USA). Headquarters (HQ) location was determined and characterized by location in California, Massachusetts, other US states, or abroad (non-US). California and Massachusetts are known biotech hotspots and had the highest numbers of company headquarters among US states. The number and nature of drug programs were assessed. Drug programs were counted if the company was considered primarily in charge of the development of a particular asset (whether through outright ownership or exclusive licensing). Minority partnerships in asset development were not considered a unique drug program. Single drugs being developed for more than one indication were counted as a single program. Drug delivery platforms were not considered a unique drug program unless coupled to a specific therapeutic agent. Medical devices were not counted. Some company websites cite development programs for numerous non-named early preclinical assets that are not otherwise specified. This scenario was ignored as they were not thought to be the main drivers of a company’s prospects for success. The number of drugs under development was stratified into the following groups: one drug, two to four drugs, and five or more drugs.

Company focus was characterized as general (nononcology), oncology, or both. Furthermore, companies were characterized according to their development of the following drug programs: gene-editing technology, RNA-focused (e.g., RNA-targeting such as antisense approaches or RNA-producing such as mRNA therapy), immunotherapy, cell therapy (such as chimeric antigen receptor-T-cell therapy), and cancer vaccines. Oncology companies were characterized as targeting either solid malignancies, hematologic malignancies, or both.

In the interest of assessing academic collaboration in the United States, funding made to physicians and teaching hospitals (academic funding), funding as reported by the Centers for Medicare & Medicaid Services, was acquired via CMS.gov Open Payments (data available from 2013 to 2018) and categorized into summed contributions over the 2013-z2018 timeframe of < or ≥$100,000. Because there was a very high correlation between companies making general payments >$100,000 (to individuals such as physicians) and making research payments >$100,000 (to academic institutions/teaching hospitals), for simplicity, these amounts were summed together into any contribution of >$100,000.

The historical company share price was obtained (StockCharts, StockCharts.com, Inc., Seattle, WA, USA) from IPO to March 9, 2019, which was the approximate 10-year anniversary of the longest bull market in history that began with the economic recovery following the Great Recession. Artificial effects from stock splits (or dividends/distributions, which were assuredly rarely applicable) were taken into account. In the interest of minimizing the impact of daily stock price fluctuation on stock performance, we used the 50-day moving average (from March 9, 2019) as the share price endpoint for statistical analysis. The opening share price of the IPO was used as the starting point for assessing performance, in lieu of the prespecified IPO price set prior to the real tradability on the open market. Due to the arbitrary nature of comparing raw stock prices between companies, we calculated the percentage change in stock price from IPO to the 50-day moving average (from March 9, 2019) as our dependent variable of interest. Stock performance was characterized as good (+ ≥25%), mediocre (±25%), or poor (- ≥25%). These cutoffs were used for being reasonable approximations of success for the investor if owning the corresponding stock and to maintain groups of similar size. While we collected for all companies intervening daily stock prices between IPO date and March 9, 2019, these were ultimately not used in this analysis due to the immense nature of the data.

Univariate and multivariate associations between factors thought to potentially impact drug success/stock performance and stock performance over time were assessed for all companies. An oncology-only subset analysis excluded companies that do not participate in oncology drug development. Chi-square tests compared categorical variables. Multivariate logistical regression tested multivariate associations. The statistical package used was SAS 9.4 (SAS Institute Inc., Cary, NC).

We also utilized the IPO list (which extended back to the year 2000, so companies with IPO before 1990 were not included) to identify defunct companies that were previously operational. These defunct companies were not included in the above analyses of stock performance, but their fate as of March 9, 2019 was characterized and analyzed. This was done to add additional descriptive information to the assessment of the likelihood of pharmaceutical company success beyond stock performance for active companies as defunct companies are likely to have had either great success (acquisition) or failure.

## Results

**Figure 1:**
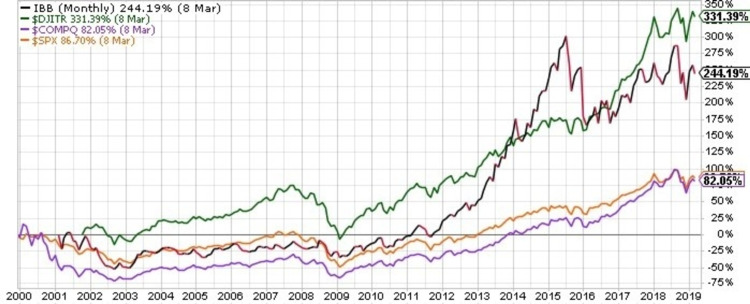
Monthly stock performance of relevant market indicators for the two decades preceding March 9, 2019. IBB is the iShares Nasdaq Biotechnology Index which tracks the biotechnology and pharmaceutical equities listed on the Nasdaq. While many of the small- and mid-cap companies included in our analysis have a small weighting in the IBB, the IBB has a notably positive performance over time principally due to good performance by large-cap companies, which have higher relative weights per company in the index. While the small- and mid-cap companies included in our analysis are much less likely to have had good performance than established large-cap pharmaceutical companies, they do often broadly trade in lock-step with the IBB on a daily basis. The IBB is presented (black and red) in comparison to the Dow Jones Industrial Average (green), the Nasdaq Composite (purple), and the Standard and Poor 500 (gold).

In the overall analysis, 420 companies were included. IPO dates ranged from January 2, 1990 to December 7, 2018. The median follow time was 7.56 years (range: 0.25-29.2). The market capitalization range was 2.916 million to 9.752 billion (mean: 848.6 million). See Figure 1 for the performance of several market indicators for the two decades preceding the endpoint of our analysis so that company performance can be taken into context.

Table 1 lists the company characteristics.

**Table 1:** Company characteristics for the overall group (n = 420)

Parameter	No. (%)
Stock exchange	
Nasdaq	404 (96.2)
New York Stock Exchange	16 (3.8)
Stock performance	
Good (+ ≥25%)	101 (24.0)
Mediocre (±25%)	77 (18.3)
Poor (- ≥25%)	242 (57.6)
Headquarters location	
Other US states	138 (32.9)
California	107 (25.4)
Abroad	88 (20.9)
Massachusetts	87 (20.8)
Pipeline focus	
Nononcology	241 (57.4)
Diverse (oncology + nononcology)	95 (22.6)
Oncology-only	84 (20.0)
Drugs under development	
1	61 (14.5)
2–4	185 (44.0)
5+	174 (41.4)
Academic funding	
	355 (84.5)
≥$100,000	65 (15.5)

Performance ranged from -99.9% to +10,451.8% (median: -42.2%). The median number of drugs in development was 5.06 (range: 1-119). The maximum summed academic contribution made between 2013 and 2018 was $227,250,297. Twenty-seven companies developed gene therapies, and 12 companies had RNA-based platforms. Of the nononcology companies, 26 focused on rare diseases, 20 focused on anti-infectives, 18 focused on neurologic conditions, four focused on ophthalmic diseases, and the remaining 173 focused on either common general medical conditions or had multiple nononcology focuses. The following variables were associated with performance in univariate analysis: IPO price (*P* < 0.001), time from IPO (*P* < 0.001), number of drug programs (*P* = 0.019), and academic funding (*P* = 0.00013), with a strong trend for diverse pipelines (*P* = 0.069). HQ location (*P* = 0.37), immunotherapy pipelines (*P* = 0.26), gene-editing pipelines (*P* = 0.31), RNA-based platforms (*P* = 0.57), cancer type targeted (solid vs. hematologic vs. both) (*P* = 0.34), CAR-T platforms (*P* = 0.80), and cancer vaccine development (*P* = 0.097) were not statistically associated with performance. On multivariate analysis (Table 2 shows odds ratios and confidence intervals), IPO price was inversely associated (*P* < 0.0001), while academic funding (*P* < 0.0001) and more drug programs (*P* = 0.0025) were positively associated with performance.

**Table 2:** Odds ratios and 95% confidence intervals assessing the relationship with good stock performance for the multivariate analysis of the overall group of small- and mid-cap pharmaceutical companies. IPO: initial public offering

Parameter	Comparators	Odds ratio	95% confidence interval
IPO opening price	Continuous variable	0.402	0.314–0.515
Time duration from IPO	Continuous variable	0.998	0.996–1.001
Academic funding ≥$100,000	Academic funding	4.290	2.378–7.741
Number of drug programs: 2–4	Single drug program	1.644	0.839–3.222
Number of drug programs: 5+	Single drug program	2.815	1.437–5.512

Within the subanalysis of oncology companies, 179 companies were included. Of the oncology companies, 69 (38.5%) targeted solid malignancies, 11 (6.1%) targeted hematologic malignancies, and the remaining 99 (55.3%) targeted both. Overall, 99 (55.3%) had immunotherapy programs, 22 (12.3%) had cell therapy programs, and 13 (7.3%) developed cancer vaccines. Oncology company distribution by stock performance was good in 45 (25.1%), mediocre in 32 (17.9%), and poor in 102 (57.0%) companies. Performance ranged from -99.9% to +10,451.8% (median: -42.0%). The following variables were associated with performance in univariate analysis: IPO price (*P* < 0.001), time from IPO (*P* < 0.001), HQ location (*P* = 0.009), diverse pipelines (*P* = 0.016), and academic funding (*P* < 0.001). Immunotherapy pipeline development had a trend for association with stock performance (*P* < 0.056). Number of drug programs (*P* = 0.14), gene-editing pipelines (*P* = 0.86), RNA-based platforms (*P* = 0.87), cancer type targeted (*P* = 0.28), cell therapy platforms (*P* = 0.78), and cancer vaccine development (*P* = 0.074) were not statistically associated with performance. On multivariate analysis (Table 3 shows odds ratios and confidence intervals), IPO price was inversely associated (*P* < 0.0001), while California HQ location (*P* < 0.01), diverse pipelines (*P* = 0.028), and academic funding (*P* = 0.0002) were positively associated with performance.

**Table 3:** Odds ratios and 95% confidence intervals assessing the relationship with good stock performance for the multivariate analysis of the oncology subgroup of small- and mid-cap pharmaceutical companies. IPO: initial public offering

Parameter	Comparators	Odds ratio	95% confidence interval
IPO opening price	Continuous variable	0.421	0.278–0.637
Time duration from IPO	Continuous variable	0.992	0.992–1.000
Academic funding ≥$100,000	Academic funding	8.842	2.788–28.035
Oncology-only pipeline	Diverse pipeline	0.468	0.237–0.922
HQ in Massachusetts	HQ in California	0.260	0.104–0.651
HQ in other US states	HQ in California	0.265	0.104–0.651
HQ abroad	HQ in California	0.631	0.256–1.555

The time duration from IPO had an inverse relationship with performance on multivariate analysis (*P* = 0.057). Immunotherapy pipeline development was not associated with performance on multivariate analysis (*P* = 0.91).

Among the full list of publicly traded companies that have undergone IPO since 2000, 388 pharmaceutical companies were captured. See Table 4 for their fates.

**Table 4:** Company fates following IPO between 2000 and 2018 (n = 388). IPO: initial public offering

Parameter	No. (%)
Active, listed on a major exchange (included in the main analysis)	231 (59.5)
Acquired (favorable)	75 (19.3)
Merger (unfavorable)	44 (11.3)
Bankrupt	21 (5.4)
Active, major exchange, share price	9 (2.3)
Active, delisted from major exchange	6 (1.5)
Taken private	2 (0.5)

## Discussion

When unencumbered by regulatory burden, principles of supply and demand suggest how much a buyer is willing to pay for a product. Health can serve as an extremely potent driver of consumer demand, leading to opportunities for great profit in the pharmaceutical industry. One argument against limiting the profitability of drug companies in the United States is that it may curb innovation; altruistic sentiment alone is likely inadequate to support the robustness currently seen in the industry. The opportunity for large financial gains for shareholders of small- and mid-cap pharmaceutical companies plays an integral role in driving the early machinery that ultimately makes new drugs available to the public. The flipside of this is that many early-stage companies gain hefty investment but fail, with no financial gain to most shareholders, and no benefit for mankind in the form of a new drug.

In this study, we have shown that it is considerably more likely that a company will either fail outright or become notably devalued following an IPO than the favorable alternative scenarios of an acquisition for a premium or a notable appreciation of stock value over time. Some factors suggest a higher likelihood of company success. Among the overall group, we found a strong positive association between a greater number of drug programs under development and stock performance. Additionally, in the overall group, we found a trend for better performance for diverse pipelines, which was statistically significant in the oncology subanalysis. These associations give validity to the commonly referenced line of thought in biotech investing that “shots-on-goal” is important. Meaning, the more opportunities the companies have for success the better, given the inherently unpredictable nature of successful randomized trials. Companies that hinge all their hopes on a single drug can be ruined by a single trial failure.

California HQ location was significantly associated with good stock performance on multivariate analysis for the oncology subset analysis. The combination of well-known academic centers nearby and a probable large pool of potential employees with biotech experience residing nearby may contribute to effective operations.

Academic funding was positively associated with stock performance in both the overall analysis and the oncology subgroup analysis. Academia likely provides constructive feedback regarding drugs under development and clinical trial structuring, as well as increased motivation for the academic entities to enroll patients in the study which can help to progress a pipeline faster. Another factor that may contribute to this finding is a sort of self-fulfilling prophecy: companies with robust cash reserves are both more likely to succeed and more likely to have additional money to spend on helpful relationships with academia. Finally, drugs and drug companies that have already shown that early successes are assuredly more likely to elicit academic support.

The finding that IPO price was inversely related to performance likely indicates that IPOs transpiring in relative market bubbles were more prone to share devaluations over time. This would suggest that investors might avoid purchasing biotech IPOs at times of exceptionally high valuations across the broader market, although conversely, for companies, this may be the ideal time to raise capital. Small- and mid-cap pharmaceutical companies often broadly trade in step with each other, often similar to other “growth” sectors across the market, and during some periods of time, the entire biotech sector may trade differently than the broader market at large. It should be noted that even within our category of good stock performance (+ ≥25%), companies may have underperformed in the broader market, depending on the percentage gain and the timeframe the stock was active. It should be noted that at the time the 50-day moving average was collected (March 9, 2019) the biotech sector, similar to the broader market at large, had been in the final phase of the choppy growth that characterized the last several years of the historically long bull market that ended with the onset of the coronavirus disease 2019 pandemic.

There are some limitations to our study. Private companies, and companies traded on lesser stock exchanges, were not included in this analysis. There are a small number of private unicorns that grow independently and become successful or are acquired by large pharmaceutical companies without ever becoming publicly traded; however, the number is low and assuredly not a substantial driver of the drugs ultimately brought to market. The likelihood of a company trading on a lesser stock exchange and ultimately bringing a blockbuster drug to market is also low. We also did not include companies that trade exclusively on foreign stock exchanges. Our analysis did not account for company share values at the intervening time points between IPO and the 50-day moving average captured on March 9, 2019, as the sheer magnitude of conducting this sort of data analysis would be beyond the scope of this manuscript. Biotech stocks are notoriously volatile, and even companies that ultimately failed or became significantly devalued likely had periods of time where investor fervor drove up share prices. Finally, value dilutions from events such as secondary public offerings, options granted to company employees, and inducement grants were difficult to take into account.

## Conclusions

The majority of included companies either outright failed or had lackluster stock performance, suggestive of low potential for drug pipeline success. High drug prices must be taken in the context of not only the value the drug brings to society but also the high probability of company failure and financial disaster inherent to the drug development process. Robust drug pipelines and close academic collaboration seem to be strongly related to success.

A common adage in investing is that the individual retail investor may benefit from investing in companies they might know the most about. For physicians and scientists, this stands to reason that pharmaceutical companies might be considered. The high likelihood of poor performance (median performance: -42%) or outright failure of these stocks (an approximate 20% rate, based on our analysis of the comprehensive list of biotech IPOs) and difficulty in predicting the outcome of binary events such as trial readouts suggests that even for investors with a theoretical edge in evaluating pharmaceutical companies, investing in small- and mid-cap pharmaceutical stocks is associated with substantially more risk than investing in a stock that more closely mimics the market at large. Nevertheless, the potential for outsized gains-29 (6.9%) of the active companies included would have been expected to substantially outperform the market, in addition to our demonstrated approximate 20% rate of acquisition for a premium-might remain alluring.
